# Upregulation of distinct collagen transcripts in post-surgery scar tissue: a study of conjunctival fibrosis

**DOI:** 10.1242/dmm.028555

**Published:** 2017-06-01

**Authors:** Li-Fong Seet, Li Zhen Toh, Stephanie W. L. Chu, Sharon N. Finger, Jocelyn L. L. Chua, Tina T. Wong

**Affiliations:** 1 Ocular Therapeutics and Drug Delivery, Singapore Eye Research Institute, 20 College Road, Singapore 169856; 2 Duke-NUS Medical School, 8 College Road, Singapore 169857; 3 Department of Ophthalmology, Yong Loo Lin School of Medicine, National University of Singapore, 5 Lower Kent Ridge Rd, National University Hospital, Singapore 119074; 4 Glaucoma Service, Singapore National Eye Centre, 11 Third Hospital Avenue, Singapore 168751; 5 School of Materials Science and Engineering, Nanyang Technological University, 11 Faculty Ave, Singapore 639977

**Keywords:** Collagen, Fibrosis, Conjunctiva

## Abstract

Excessive accumulation of collagen is often used to assess the development of fibrosis. This study aims to identify collagen genes that define fibrosis in the conjunctiva following glaucoma filtration surgery (GFS). Using the mouse model of GFS, we have identified collagen transcripts that were upregulated in the fibrotic phase of wound healing via RNA-seq. The collagen transcripts that were increased the most were encoded by *Col8a1*, *Col11a1* and *Col8a2*. Further analysis of the *Col8a1*, *Col11a1* and *Col8a2* transcripts revealed their increase by 67-, 54- and 18-fold, respectively, in the fibrotic phase, compared with 12-fold for *Col1a1*, the most commonly evaluated collagen gene for fibrosis. However, only type I collagen was significantly upregulated at the protein level in the fibrotic phase. Type VIII and type I collagens colocalized in fibrous structures and in ACTA2-positive pericytes, and appeared to compensate for each other in expression levels. Type XI collagen showed low colocalization with both type VIII and type I collagens but can be found in association with macrophages. Furthermore, we show that both mouse and human conjunctival fibroblasts expressed elevated levels of the most highly expressed collagen genes in response to TGFβ2 treatment. Importantly, conjunctival tissues from individuals whose GF surgeries have failed due to scarring showed 3.60- and 2.78-fold increases in type VIII and I collagen transcripts, respectively, compared with those from individuals with no prior surgeries. These data demonstrate that distinct collagen transcripts are expressed at high levels in the conjunctiva after surgery and their unique expression profiles may imply differential influences on the fibrotic outcome.

## Abstract (precis)

**Summary:** As well as providing an objective quantitative measure, distinct collagen genes may further aid in the characterization and definition of the development of fibrosis.

## INTRODUCTION

Fibrosis, or scarring when it occurs in response to injury, is commonly defined as the development of excessive fibrous connective tissue. As collagens are the major fibrous proteins found in connective tissue (Muiznieks and Keeley, 2013), the measurement or visualization of collagen deposition is commonly used to assess fibrosis in a myriad of disorders involving the heart (Lijnen et al., 2012), liver (Liu et al., 2012) and kidney (Farris and Colvin, 2012), as well as complex multi-system diseases such as scleroderma (Balbir-Gurman and Braun-Moscovici, 2012). Type I collagen, which is the major component of extracellular matrix (ECM) and the most abundant form of collagen in the body, is the most frequently measured collagen component of scar proteins.

Collagen deposition is used to evaluate surgical failure in glaucoma filtration surgery (GFS), also known as trabeculectomy. GFS is performed to lower high intraocular pressure, a major risk factor associated with the development and progression of glaucoma (Sawchyn and Slabaugh, 2016). GFS increases the outflow of fluid from the eye via a sclerostomy. However, GFS can fail, due primarily to subconjunctival and episcleral fibrosis, which develops over the sclerostomy site (Radcliffe, 2010). Collagen accumulation, as an indicator of scarring, is generally evaluated via visualization of histochemically stained tissue sections in studies of GFS outcomes in humans (Molteno et al., 2013) and experimental large animal models of GFS (SooHoo et al., 2012; Ekinci et al., 2014). This approach is far from being quantitative, given the potential variability in staining methods, imaging quality, number of sections analysed and selection of areas for examination.

The development of a mouse model of GFS (Seet et al., 2010) enables a quantitative assessment of fibrotic development through molecular analyses (Seet et al., 2013). This mouse model responded to mitomycin C (MMC), a commonly applied adjunctive antifibrotic therapy in clinical GFS, in a similar manner to that observed in patients (Seet et al., 2011). The fibrotic phase in this mouse model, which is measurable on day 7 post-experimental surgery, is marked by increased expression of extracellular matrix genes, including type I collagen (*Col1a1*) (Seet et al., 2013). *Col1a1* expression, as a quantitative marker for conjunctival fibrosis, can be measured at both the mRNA and protein levels via real-time quantitative polymerase chain reaction (qPCR) and immunoblotting techniques in the mouse model. Both techniques are independently quantitative and have the capacity to assess the entire scarring area (Seet et al., 2013).

Besides type I collagen, other collagen genes may potentially provide complementary signals for the development of fibrosis in the post-surgical conjunctival tissue. An unbiased, global discovery of these genes is not feasible with human subjects as conjunctival biopsies, if taken from patients whose surgeries have failed due to fibrosis, would be heterogeneous, having been previously exposed to a variety of anti-hypertensive and anti-fibrotic drug therapies. However, with the help of the mouse model of GFS, we were able to uncover additional collagen genes that may refine our understanding of the development of fibrosis in the post-operative conjunctiva. We performed transcriptome profiling of the wound-healing phases to reveal the identities of collagen genes that were induced in fibrosis. Localization of the top-ranked collagen genes in fibrotic phase tissues demonstrated potentially unique roles of these proteins in fibrosis. We also ascertained that the top-ranked collagen transcripts were induced in response to profibrogenic TGFβ2 in conjunctival fibroblasts, which are the main effector cells implicated in eliciting the fibrotic response in GFS (Hitchings and Grierson, 1983; Skuta and Parrish, 1987; Jampel et al., 1988). We further confirmed the human relevance of the top-ranked type VIII collagen by analyses of human Tenon's fibroblasts, as well as through comparison of conjunctival tissues obtained from patients whose previous surgeries have failed against those with no prior GFS. Taken together, we demonstrate that scarring in GFS is characterized by the high induction of selective collagen transcripts with potentially distinct roles in fibrosis.

## RESULTS

### Expression of distinct collagen transcripts in the late phase of conjunctival wound healing

**Fig. 1.**
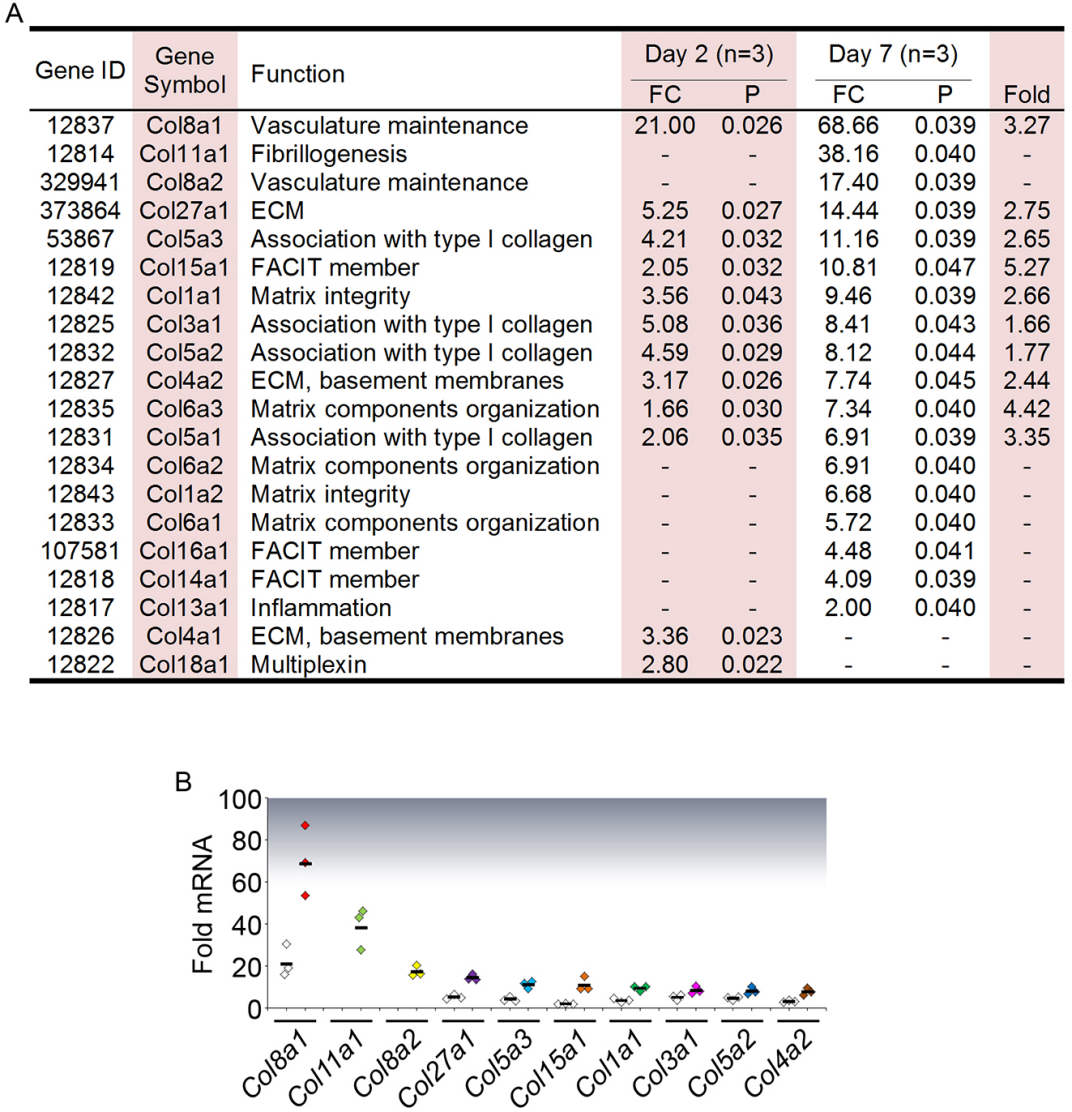
**Upregulated collagen transcripts in the day 2 and day 7 post-experimental GFS transcriptomes.** (A) Collagen genes are ranked in descending order from the highest to lowest transcript induction on day 7. Three paired groups at each time point were analysed (*n*=3). Each paired group consisted of 10 operated tissues from the left eyes pooled together for comparison with 10 unoperated tissues from the contralateral right eyes similarly pooled together. FC, mean fold-change of three groups comparing operated against paired unoperated conjunctival tissues. *P* values shown have been adjusted using the Benjamini-Hochberg False Discovery Rate (FDR) method. Fold, fold-change between day 7 and day 2 (day 7 FC/day 2 FC); -, *P*>0.05 (not significant); ECM, extracellular matrix; FACIT, fibril-associated collagens with interrupted helices. (B) The 10 collagen transcripts that were expressed at the highest levels in the day 2- and day 7- operated conjunctival transcriptomes, calculated relative to the unoperated counterparts (*n*=3). Day 2 expression is represented by white diamonds, whereas day 7 expression is represented by coloured diamonds. Day 2 expression levels that were not significantly different from the respective unoperated baselines are not presented in the plot.

To determine the gene expression profiles in the early and fibrotic phases of wound healing in GFS, we performed experimental surgery in the mouse and analysed the day 2 and day 7 operated tissues by high-throughput next-generation mRNA sequencing. Eighteen collagen genes were significantly upregulated in the late-phase transcriptome by at least twofold relative to unoperated tissues (Fig. 1A). The three collagen transcripts that were overexpressed the most in the fibrotic phase were *Col8a1*, *Col11a1* and *Col8a2*, while *Col1a1* transcript was ranked 7th (Fig. 1B).

**Fig. 2.**
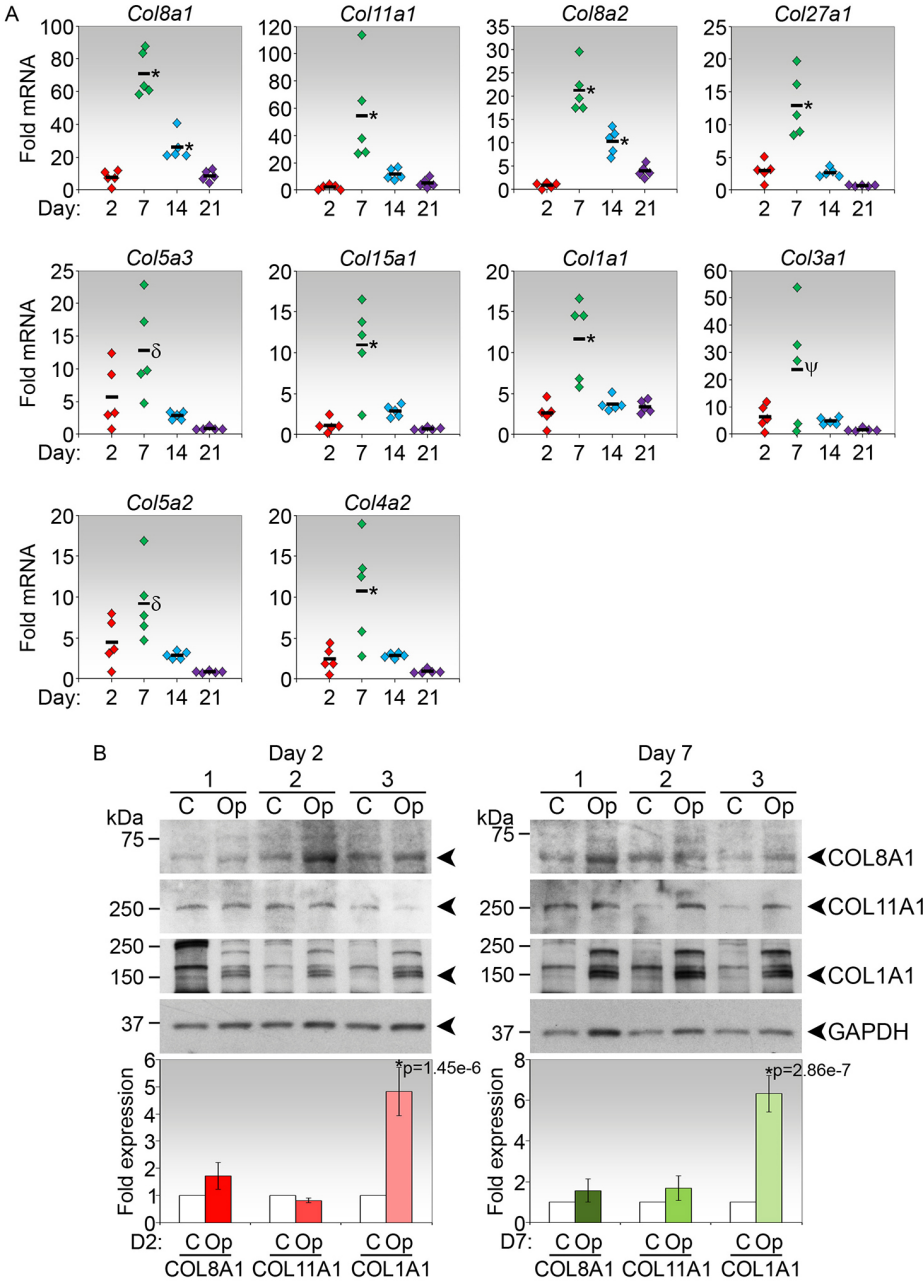
**Verification of collagen transcripts that were upregulated the most in the late phase of conjunctival wound healing by qPCR.** (A) Real-time PCR analyses of the 10 most highly expressed collagen genes in mouse conjunctival tissues harvested 2, 7, 14 and 21 days after experimental surgery. Data are calculated as fold-change relative to paired unoperated controls. The mean fold-change of five groups, each consisting of operated tissues pooled from three mice, for each time point is indicated (*n*=5). *P<0.05 for fold-change compared with all other time points; ^δ^*P*<0.05 for fold-change on day 7 compared with both days 14 and 21; ^ψ^*P*<0.05 for fold-change on day 7 compared with day 21 only. (B) Immunoblot analyses of COL8A1, COL11A1 and COL1A1 in mouse conjunctival tissues harvested on day 2 and day 7 post-experimental surgery. Three paired groups for each time point are shown (*n*=3). Op, operated tissues pooled from five independent eyes per group; C, paired untreated controls pooled similarly. Fold-change in expression in operated relative to control tissues, both normalized to GAPDH, and the associated *P* values corrected by Bonferroni adjustment, where significant, are shown below the respective immunoblot.

The ten top-ranked collagen transcripts in the late-phase transcriptome were further verified by real-time PCR. Similar expression profiles were observed for the day 2 and day 7 time points (Fig. 2A). Additional examination of the mRNA expression profiles at the later time points of days 14 and 21 post-surgery revealed that expression of the collagen genes in the operated conjunctival tissues generally peak on day 7, where mean fold increases from the unoperated baseline levels were significantly higher than the mean fold increases at all the other time points (Fig. 2A). These data suggest that an active increase in expression of collagen genes in the operated tissue is likely to peak in the first week after surgery followed by a return to normal expression thereafter.

The expression of the top three collagen genes, including type I collagen, was also evaluated at the protein level. As *Col8a1* and *Col8a2* encode the two polypeptide subunits of type VIII collagen, we analysed COL8A1 as representative of type VIII collagen protein expression. Using immunoblotting, we observed that only type I collagen was significantly induced on both day 2 and day 7 following experimental surgery when compared with their respective unoperated controls (Fig. 2B). Hence, type I collagen remains the most stably induced collagen protein in the late phase of conjunctival wound healing (Fig. 2B).

### Differential localization of COL8A1, COL11A1 and COL1A1 in the fibrotic phase of conjunctival wound healing

**Fig. 3.**
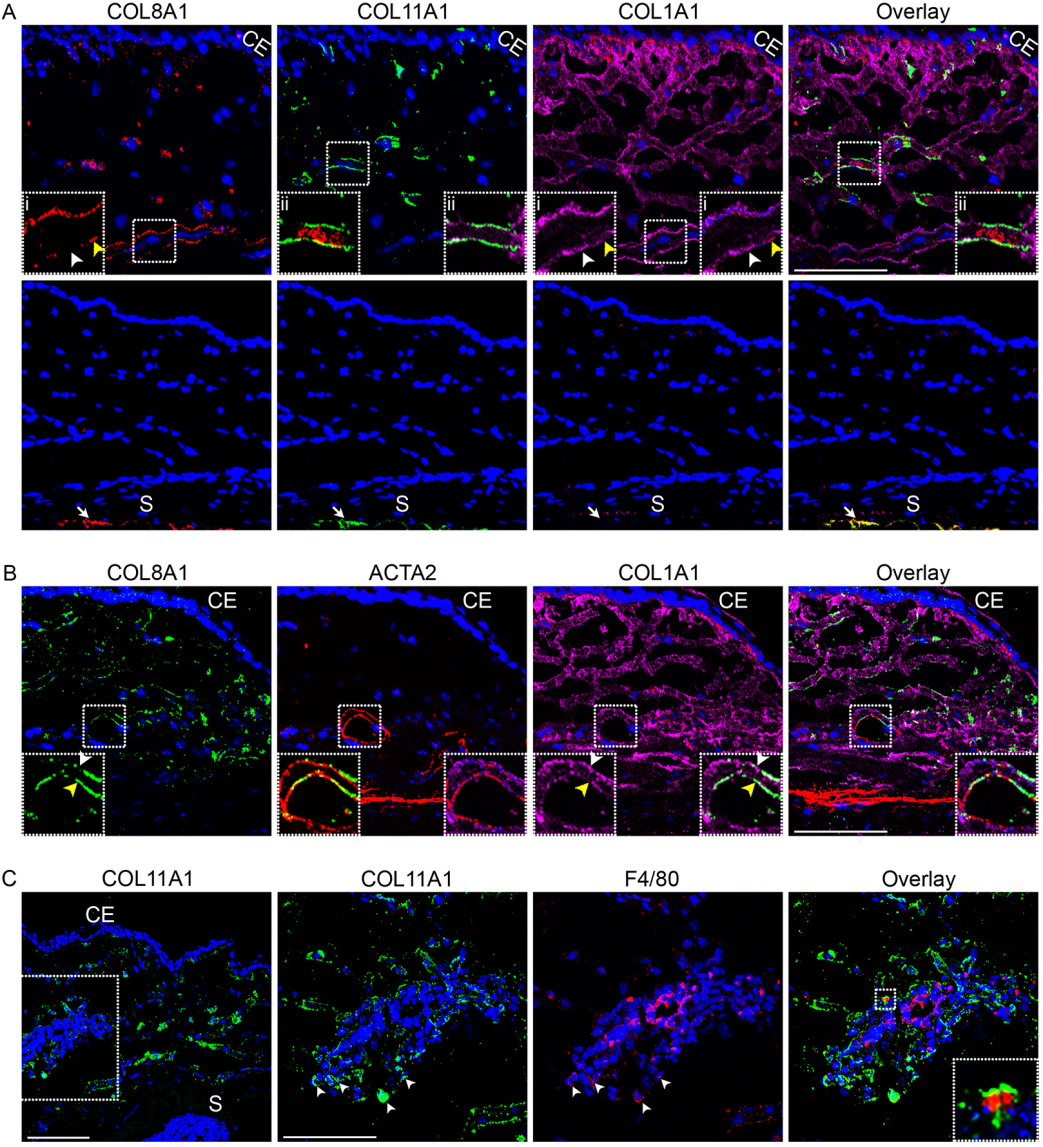
**Immunolocalization of COL8A1, COL11A1 and COL1A1 in the day 7-operated mouse conjunctival tissue.** (A) Co-immunolabelling of COL8A1 (red), COL11A1 (green) and COL1A1 (magenta). Upper panels, insets (i) show magnified images of the boxed areas in the first and third images, with yellow and white arrowheads indicating high COL8A1/low COL1A1 and low COL8A1/high COL1A1 co-immunolabellings, respectively; inset (ii) left, second image, shows a magnified image of the boxed area co-immunolabelled for COL8A1 and COL11A1; inset (ii) right, second image, shows a magnified image of the boxed area co-immunolabelled for COL11A1 and COL1A1; inset (ii), fourth image, shows a magnified overlay image of the boxed area co-immunolabelled for all three collagens. None of the insets includes DAPI staining. Lower panels, a section from the same eye incubated with only the secondary antibodies used in the upper panel showed minimum non-specific staining in the bleb area in comparison with non-specific staining, which was observable in the choroid (arrow). S, sclera. (B) Co-immunolocalization of COL8A1 (green) and COL1A1 (magenta) with ACTA2 (red). Yellow and white arrowheads in insets indicate COL8A1 and COL1A1 co-immunolabelling patterns, as indicated in A. Co-immunolabelling of ACTA2 with COL8A1 (left inset, second image) or COL1A1 (right inset, second image) appears yellow or blue, respectively. Co-immunolabelling of COL8A1 and COL1A1 appears white (right inset, third image). None of the insets includes DAPI staining. (C) Co-immunolocalization of COL11A1 (green) with F4/80+ cells (red). Inset, fourth image, shows a magnified overlay image of the boxed area co-immunolabelled for COL11A1 and F4/80. All pictures were captured by confocal microscopy. Scale bars: 100 μm.

To determine potentially unique roles of type VIII, type XI and type I collagen in the fibrotic phase of conjunctival wound healing, we examined their localization in triple-immunostained tissues. At first glance, COL8A1 seemed to colocalize with COL1A1, particularly in association with long fibrous structures (Fig. 3A). On closer examination, it can be discerned that COL8A1 expression appeared high where COL1A1 was detected at very low levels (Fig. 3A,B) and vice versa (Fig. 3A,B). By contrast, COL11A1 appeared to have very little colocalization with COL1A1. Interestingly, COL11A1 expression was adjunctive to that of COL8A1, appearing to localize in the periphery of areas where COL8A1 was detected (Fig. 3A).

To probe whether COL8A1 is present in pericytes surrounding vascular endothelial cells, we co-immunostained the day 7 operated tissues to detect α-smooth muscle actin (ACTA2) expression (Skalli et al., 1989). COL8A1 was observed to partially colocalize with ACTA2, as did COL1A1 (Fig. 3B). Again, COL8A1 appeared to be expressed at higher levels in ACTA2-positive areas where COL1A1 expression was apparently low in expression (Fig. 3B) and vice versa (Fig. 3B). These observations suggest that both COL8A1 and COL1A1 are components of pericytes that wrap around vascular endothelial cells.

As COL11A1 appeared to be mainly expressed in non-fibrous structures in the fibrotic phase, we probed the possibility that this collagen may be associated with pro-inflammatory cells such as macrophages. In areas heavily populated with cells, including F4/80+ macrophages, COL11A1 was also present and could be detected in association with macrophages (Fig. 3C). Taken together, these data suggest that COL8A1, COL11A1 and COL1A1 have unique localizations and may perform different functions in the fibrotic process in the post-surgical conjunctiva.

### Induction of collagens by profibrotic TGFβ2 in conjunctival fibroblasts

**Fig. 4.**
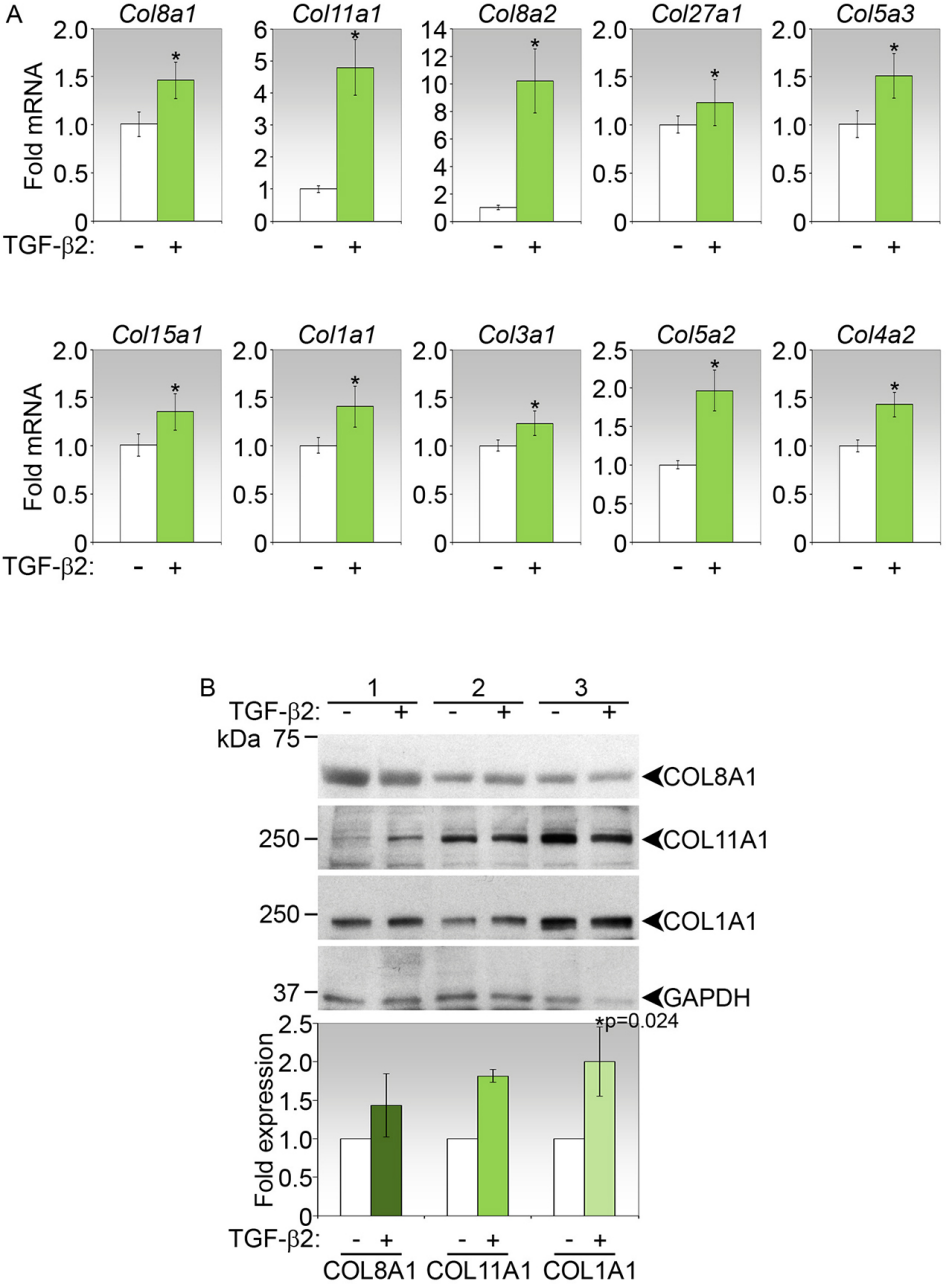
**Induction of top collagen genes by TGFβ2 in primary mouse conjunctival fibroblasts.** (A) Real-time PCR analyses of collagen genes in fibroblasts stimulated for 72 h with TGFβ2. Data shown are representative of three independent experiments, and show mean fold-change±s.d. relative to untreated controls. *P<0.05 for fold-change in treated versus control. (B) Immunoblot analyses of COL8A1, COL11A1 and COL1A1 in mouse fibroblasts treated as indicated for 72 h. Three independent sets of experiments are shown. Fold-change in expression in TGFβ2-treated relative to control cells, both normalized to GAPDH, and *P* value (corrected by Bonferroni adjustment) where significant, are shown in the densitometric analyses below the immunoblot.

As TGFβ2 is likely to play important roles during late-phase fibrosis (Seet et al., 2013), we next investigated the capacity of this cytokine to induce the expression of the top ten collagen genes in primary conjunctival fibroblasts. The cells responded to TGFβ2 by increasing the expression of all the collagen genes examined at the mRNA level (Fig. 4A). However, the profiles of induction of the top three collagen transcripts by TGFβ2 did not mirror that observed in the operated conjunctival tissues in the fibrotic phase of wound healing (Fig. 2A). In the mouse conjunctival fibroblasts, *Col8a2* transcripts were the most highly induced, followed by *Col11a1*, whereas the remaining collagen mRNAs were generally induced by twofold or less (Fig. 4A). At the protein level, only COL1A1 was significantly elevated by TGFβ2 treatment (Fig. 4B).

### Induction of collagens in human conjunctival fibroblasts by TGFβ2 and in tissues of individuals requiring repeat surgical procedure

**Fig. 5.**
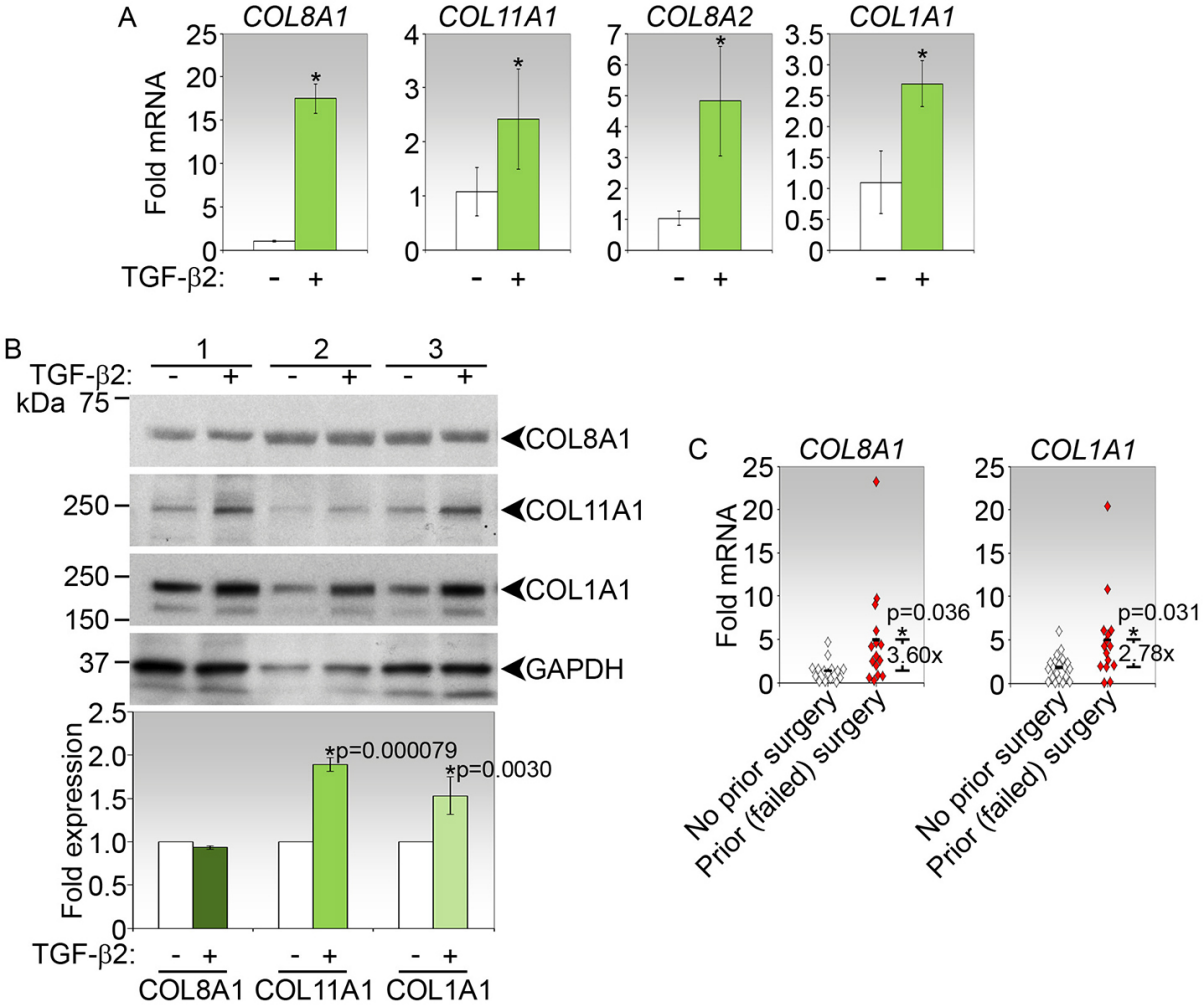
**Induction of top collagen genes by TGFβ2 in primary human conjunctival fibroblasts and in individuals with surgical failure.** (A) Real-time PCR analyses of *COL8A1*, *COL11A1*, *COL8A2* and *COL1A1* in primary human conjunctival fibroblasts stimulated with TGFβ2 for 72 h. Data shown are representative of three independent experiments, and calculated as mean fold-change±s.d. relative to untreated controls. *P<0.05 for fold-change in treated versus control. (B) Immunoblot analyses of COL8A1, COL11A1 and COL1A1 in human fibroblasts treated as indicated for 72 h. Three independent sets of experiments are shown. Fold-change in expression in TGFβ2-treated relative to control cells, both normalized to GAPDH, and *P* values (corrected by Bonferroni adjustment) where significant, are shown in the densitometric analyses below the immunoblot. (C) Real-time PCR analyses of *COL8A1* and *COL1A1* in human conjunctival tissues from individuals requiring repeat surgeries (*n*=15) relative to those with no prior GFS (*n*=20). Data for *COL8A1* in tissues of individuals with no prior surgeries was only available from 15 patients as five samples did not produce detectable values. Data shown are calculated as fold-change in expression in tissues of repeat surgery patients relative to that from patients with no prior surgery. Significant fold-changes between the two groups of patients and the associated *P* values (*) are indicated. Each symbol represents one patient.

To determine the relevance of the collagen genes for detecting fibrosis in human tissues, we measured the response of primary human conjunctival fibroblasts to TGFβ2 stimulation. The three most highly expressed collagen genes in the mouse transcriptome as well as *COL1A1* were significantly induced in the presence of TGFβ2 at the mRNA level (Fig. 5A). However, the profile in the human cells did not exactly resemble that in mouse tissues or their cells. In human conjunctival fibroblasts, *COL8A1* transcript was the most highly induced by TGFβ2, followed by *COL8A2* and, finally, *COL11A1* mRNAs (Fig. 5A). At the protein level, both COL11A1 and COL1A1 levels were significantly increased by TGFβ2 treatment (Fig. 5B).

We further determined the expression of collagen transcripts in the conjunctiva of glaucoma patients who required GFS treatment. The quantity of conjunctival tissues that can be retrieved from patients undergoing GFS is extremely limited and sufficient only for the analysis of a few genes. We therefore prioritized the analyses of top-ranked *COL8A1*, as well as *COL1A1*, the most commonly analysed gene. The transcript levels of these two collagen genes were measured in conjunctival tissues from patients who had undergone prior surgeries that had failed due to scarring and from those who had not undergone the procedure before. Tissues were obtainable from the former group of patients as repeat surgery is used to manage a previous filtration surgery that has failed due to fibrosis (Radcliffe, 2010). *COL8A1* and *COL1A1* mRNAs were significantly increased in the conjunctival tissues from patients whose prior surgeries had failed compared with those from patients who had not previously undergone surgery by 3.60- and 2.78-fold, respectively (Fig. 5C). The elevation of two transcripts may therefore be indicative of excessive scarring after surgery which may contribute to surgery failure. Taken together, these data support the findings in the mouse model that the top-ranked collagen transcripts may also contribute to the profibrotic response in patients.

## DISCUSSION

To our knowledge, this is the first study that reveals the potential involvement of distinct collagen genes in fibrosis after GFS. By transcriptome profiling of the wound-healing response in the mouse model of GFS, we have identified type VIII collagen as the top-ranked upregulated gene followed by type XI collagen. Conjunctival tissues from glaucoma patients whose surgeries have failed also registered increased type I and type VIII collagen transcripts, supporting the relevance of these collagens in the development of fibrosis after GFS.

The contribution of type VIII collagen to tissue fibrosis is unclear. Type VIII collagen is a member of the short-chain non-fibrillar collagen family and comprises α1 and α2 chains, both found in our study to represent the highest and third highest, respectively, upregulated collagen transcripts following experimental surgery. Type VIII collagen may be produced by macrophages and smooth muscle cells, and is inducible by growth factors and cytokines (Sibinga et al., 1997; Plenz et al., 2003; Cherepanova et al., 2009; Garvey et al., 2010). Upregulation of type VIII collagen was observed in kidney biopsies from individuals with diabetic nephropathy where tubulointerstitial fibrosis is a pathological hallmark (Gerth et al., 2007). Conversely, lack of type VIII collagen conferred renoprotection in a mouse model of diabetic nephropathy (Hopfer et al., 2009), modulated TGFβ1 signalling (Loeffler et al., 2011) and resulted in abnormal development of the anterior segment of the eye, including the cornea (Hopfer et al., 2005). Defects in vessel wall thickening and remodelling, as well as fibrous cap formation in the injured femoral artery, a condition associated with reduced fibrillar type I collagen accumulation, were also noted (Lopes et al., 2013). We too observed close association of type VIII with type I collagen in the operated conjunctival tissue, especially in pericytes that make up the outer walls of blood vessels, supporting a role for the two collagens in blood vessel maturation in the fibrotic phase of the operated tissue (Bergers and Song, 2005). The two collagens seemingly substituted for each other when either expression was low, suggesting that an intimate supportive interaction exists between the two. Determining whether type I and VIII collagens serve unique or redundant roles in this context requires further experimentation. Given that type VIII collagen is reported to be associated with other collagens, elastin, microfibrillar proteins and proteoglycans (Sawada and Konomi, 1991; Sutmuller et al., 1997), a role for type VIII collagen in matrix assembly seems likely. The relatively lower protein induction of type VIII collagen compared with type I collagen in the operated tissues in the fibrotic phase of wound healing may suggest that type VIII collagen is highly potent, and therefore highly regulated at either the translational level and/or subject to protease digestion in the tissue environment undergoing active wound healing. Indeed, type VIII collagen has been shown to be rapidly degraded by neutrophil elastase, in apparent contrast to type I collagen, which was resistant (Kittelberger et al., 1992). Hence, we believe that ultra-high upregulation of type VIII collagen at the transcript level, but not the protein, is a more accurate indicator of the fibrotic phase response.

The contribution of type XI collagen to tissue fibrosis is equally nebulous. Like type I collagen, type XI collagen belongs to the fibril-forming group. However, while type I collagen is considered to be a major fibrillar collagen, type XI collagen is a minor fibrillar collagen based on abundance in the collagen fibrils of normal tissues (Shoulders and Rainez, 2009). Although type XI collagen is quantitatively a minor collagen, it is known to co-assemble with collagen I or II to form heterotypic fibrils in a regulated tissue-specific manner (Birk and Bruckner, 2011) and to have been implicated in the regulation of fibril assembly by nucleating fibril formation (Wenstrup et al., 2011) and/or regulation of collagen fibril diameter. The latter is supported by observations that mice carrying homozygous mutations for *Col11a1* have bone defects associated with thickened and shorter cartilage collagen fibrils (Seegmiller et al., 1971; Li et al., 1995; Fernandes et al., 2007). Similarly, mutations in *COL11A1* in humans caused fibrochondrogenesis, a potentially lethal disorder (Tompson et al., 2010; Akawi et al., 2012; Hufnagel et al., 2014). Mutations in the *COL11A1* gene are also associated with the autosomal dominant type II Stickler syndrome, which features a variable phenotype, including visual disturbances such as type 2 vitreous anomaly, childhood-onset myopia, glaucoma, cataracts and retinal detachment (Snead et al., 1996; Richards et al., 2013). Recent reports have established an association between *Col11a1* expression and cancer, a process that encompasses many parallels with wound healing (Schäfer and Werner, 2008). Upregulation of *COL11A1* has been described in several cancers, including colorectal (Fischer et al., 2001), breast (Freire et al., 2014) and non-small cell lung cancer (Chong et al., 2006). In head and neck squamous cell carcinoma cells, *COL11A1* facilitated their proliferation, migration and invasion (Sok et al., 2013). In ovarian cancer, *COL11A1* mRNA levels correlated with disease progression and survival, and its expression is thought to mediate tumour invasiveness (Wu et al., 2014). One speculation is that type XI collagen may facilitate tumour metastasis by promoting fibrillogenesis, as collagen is known to play an integral role in tumour metastasis (Zhang et al., 2013). In the operated mouse conjunctiva, we did not observe an obvious colocalization between type I and XI collagens. However, we did observe an association between type XI collagen and F4/80+ macrophages in the operated conjunctival tissue, suggesting that this collagen may be induced in macrophages that participate in the wound-healing process. Macrophages are known to express virtually all known collagen genes (Schnoor et al., 2008). As with type VIII collagen, the protein levels of type XI collagen in the late-phase operated tissues did not reflect the high transcript expression. Again, this may be due to type XI collagen being susceptible to degradation by proteases such as cathepsin (Maciewicz et al., 1990), which is expressed in the eye (Wassélius et al., 2003). Hence, type XI collagen may also be highly regulated at the protein level, which may be vital for reining in its activity if it has potent effects on fibrosis.

TGFβ is a well-established profibrotic cytokine that is implicated in mediating fibrosis in the mouse model of GFS (Seet et al., 2013) and GFS failure in patients (Saika, 2006; CAT-152 0102 Trabeculectomy study group et al., 2007). Although the listed collagen genes were induced in mouse conjunctival fibroblasts upon stimulation with TGFβ2, the profiles were not similar to those observed in the operated tissues. One possible explanation may be that conjunctival fibroblasts, which reliably induce type I collagen upon stimulation, are not principal producers of other collagen types. The association of type VIII and type XI collagens with pericytes and macrophages is a strong indication that other cell types are involved in producing these collagens. Indeed, type VIII collagen induction was associated with stimulated vascular smooth muscle cells (Sibinga et al., 1997). Moreover, other profibrogenic agonists other than TGFβ2 are expected to play important roles in inducing these collagen genes in fibroblasts and other cell types *in vivo*. Hence, where expression of collagen genes is concerned, it may not be possible to accurately extrapolate *in vitro* behaviour based on a single cell type or agonist to the *in vivo* tissue response involving many cell types and profibrogenic factors simultaneously.

Collectively, we have identified additional collagen genes in addition the commonly measured type I collagen that were expressed at high levels in the mouse and human conjunctiva after GFS. It is potentially possible to derive a quantitative correlation between the elevation of these transcripts and the severity of fibrosis to identify patients at risk for surgery failure. Moreover, their high inductions and unique expression profiles may reveal variations in the scarring process between surgery repeats, paving the way for more effective customized treatment. Furthermore, as glaucoma surgery is often performed in the presence of antifibrotic agents (Radcliffe, 2010), these collagens may also serve as indicators of the effectiveness and efficiency of alternative adjunctive anti-fibrotic therapies, and perhaps even throw light on the mechanisms involved.

## MATERIALS AND METHODS

### Mouse model of GFS

All experiments with animals were approved by the Institutional Animal Care and Use Committee (IACUC) and treated in accordance with the Association for Research in Vision and Ophthalmology (ARVO) Statement on the Use of Animals in Ophthalmic and Vision Research. Eight- to 10-week-old male and female 129SVE mice were used to validate the RNA-seq results. Experimental surgery was performed as described previously on one eye while the contralateral unoperated eye was used as baseline for comparison (Seet et al., 2010). Operated and unoperated tissues were harvested at the indicated time points, with day 2 and day 7 representing the early and fibrotic phases of wound healing, respectively (Seet et al., 2013).

### Human subjects

Patients were recruited from the glaucoma specialist clinics at the Singapore National Eye Centre between 2012 and 2014. This study was reviewed and approved by the institutional review and ethics board at the Singapore Eye Research Institute (SERI) and adhered to the tenets of the Declaration of Helsinki. Written informed consent was obtained from all subjects. Thirty-five eyes with medically uncontrolled primary glaucoma (primary open angle and primary closed angle glaucoma) demonstrating poorly controlled intraocular pressure (IOP) despite maximal medical therapy and/or progressive visual field loss and optic disc cupping, and requiring a trabeculectomy or phacotrabeculectomy, were recruited. A medically uncontrolled primary glaucoma is defined as an IOP of >21 mmHg, despite maximal medical therapy in eyes with primary open angle or angle closure glaucoma. Of these 35 eyes, 20 had no prior trabeculectomy surgery, while the other 15 eyes had a prior failed trabeculectomy. Prior trabeculectomy was performed using mitomycin C (0.4 mg/ml)-soaked sponges applied intraoperatively in the subconjunctival space of the surgical site. The sponges were removed after 2 min followed by copious irrigation with 50 ml of balanced salt solution in the area treated with mitomycin C. If the eye was pseudophakic, the cataract surgery was performed at least 6 months prior to study recruitment. Prior to collecting the conjunctival tissue, the eye to be operated on was cleaned and draped. A partial thickness clear corneal 7/0 traction vicryl suture was placed for good surgical exposure of the superior conjunctival space. A superior conjunctival peritomy was performed. A small amount of tenon tissue was then obtained from the site of conjunctival dissection and stored in RNAlater buffer (Thermo Fisher Scientific) and stored at −80^o^C prior to analysis.

### Primary conjunctival fibroblast cell culture

Primary human conjunctival fibroblasts were cultured from cadaver conjunctival samples. Cadaver eyes with no known prior history of glaucoma were procured from the Lions Eye Institute for Transplant and Research (Tampa, FL, USA) with written consent from the next of kin and adherence to the principles outlined in the Declaration of Helsinki. Primary mouse conjunctival fibroblasts were cultured from the eyes of 129SVE mice. The cells were cultured as described previously (Seet et al., 2012, 2013). Cells of passage eight or under were used. Treatment with TGFβ2 (100-35B-5; PeproTech) was performed at 8 ng/ml for 72 h. Three repeats using independent primary cultures were performed for each *in vitro* experiment.

### RNA sequencing and data analysis

Left, operated eyes of ten mice were pooled into one group, in a total of three groups per time point. The right, unoperated control eyes were similarly pooled as baselines for pair-wise comparisons. Tissues were harvested on either day 2 or day 7 post-surgery in RNAlater buffer (Thermo Fisher Scientific) and lysed by freezing in liquid nitrogen followed by grinding with a micropestle for several cycles. The lysed tissue was then resuspended in RNeasy Lysis buffer (Qiagen) and further lysed by passing through a 23-gauge needle at least 10 times. Total RNA was then purified using the RNeasy kit (Qiagen). RNA samples were assessed with the Bioanalyser RNA 6000 nano kit (Agilent Technologies) for RNA integrity and Nanodrop (Thermo Fisher Scientific) for RNA quality. Good quality total RNA (1 μg per sample) was used and each library was tagged with unique indices for subsequent multiplex sequencing. The size distribution of the final indexed libraries was checked using the Agilent Technologies DNA 1000 chip (Agilent Technologies). Sequencing was performed by Yourgene Bioscience using Illumina HiSeq2500. The samples were multiplexed and sequenced using 100 bp paired-end processing.

Trimming of adapter from raw reads was performed with Trim Galore version 0.3.7. The trimmed data were mapped or aligned to the mouse genome using Tophat2 version 2.0.9 aligner against the UCSC Reference sequence database (mm10). Gene expression data, mapped and filtered, was quantitated using Strand NGS 2.0. Normalization of read count values was applied using the DESeq package. At least 100 raw reads in all samples was the cut-off threshold for consideration in the analysis. A statistical test comprising *t*-test with Benjamini-Hochberg False Discovery Rate (FDR) (Benjamini and Hochberg, 1995) was performed and differentially expressed genes (*P*<0.05) were identified. A fold-change analysis was then performed to identify differentially expressed genes with a more than 2-fold change.

### Real-time quantitative PCR analysis (qPCR)

Both mouse and human conjunctival fibroblasts and tissues were processed and analysed by qPCR, as described previously (Seet et al., 2012, 2013). For verification of RNA-seq data, left operated eyes of three mice were pooled into one group, in a total of five groups per time point. The right unoperated control eyes were similarly pooled as baselines for pair-wise comparisons.

All samples were amplified by qPCR in triplicate. All mRNA levels were measured as C_T_ threshold levels. The best housekeeping gene for each experimental condition was determined using the NormFinder software (Andersen et al., 2004). The expression levels of collagen genes from mouse operated conjunctival tissues were normalized to the expression level of 18S rRNA. The expression levels of collagen genes from mouse and human conjunctival fibroblasts, as well as human conjunctival tissues, were normalized to the expression level of *Rpl13a*. Values were calculated as fold-change by the 2^−ΔΔCT^ method. Primer sequences are shown in Table 1.

**Table 1.**
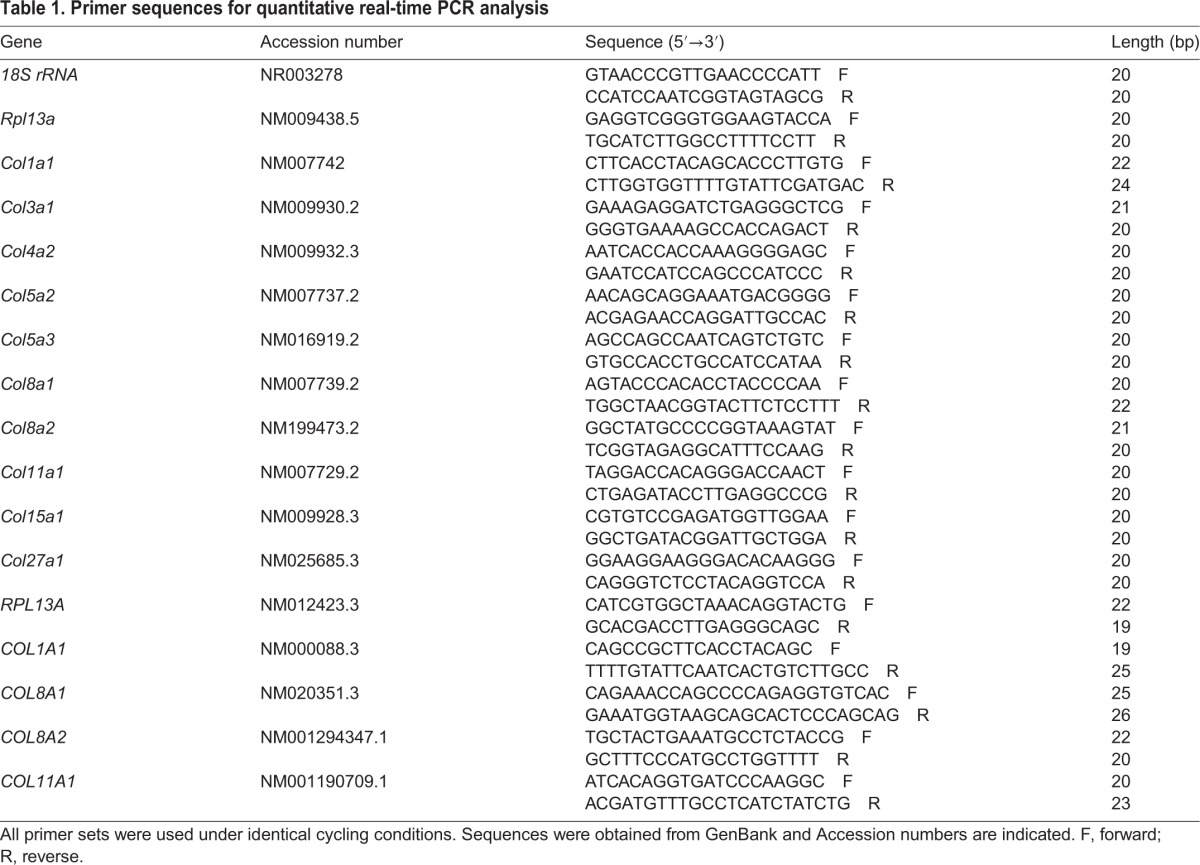
**Primer sequences for quantitative real-time PCR analysis**

### Immunoblotting

For analyses of post-operative response on day 2 and day 7, the left operated eyes of five mice were pooled into one group, in a total of three groups per time point. The right unoperated control eyes were similarly pooled for pair-wise comparisons. Tissues were harvested and processed as described previously (Seet et al., 2013). For cultured primary human and mouse conjunctival fibroblasts, three sets of independent experiments were performed. Untreated control and TGFβ2-treated cells were harvested and processed as mentioned previously (Seet et al., 2012). Tissue and whole-cell lysates were resolved by SDS-polyacrylamide gel electrophoresis followed by immunoblotting as previously described (Seet et al., 2013). For cultured cells, anti-type I (1:2000; H00001277-M01), -type VIII (1:500; 17251-1-AP) and -type XI (1:4000; NBP1-98463) collagen antibodies used were from Abnova, ProteinTech Group and Novus Biologicals, respectively. For mouse tissues, anti-type VIII collagen (1:2000; sc-99356) obtained from Santa Cruz Biotechnology was used. Horseradish peroxidase (HRP)-conjugated secondary antibodies were from Jackson Immunoresearch Laboratories. Densitometric analyses, where potential errors in loading were corrected to levels of the housekeeping GAPDH, was performed as reported previously (Seet et al., 2013).

### Immunofluorescence

Immunostaining of day 7-operated eye cryosections was performed as described previously (Seet et al., 2010). Co-immunolabellings by antibodies against type I collagen from Abnova (1:20; H00001277-M01), type VIII collagen from ProteinTech Group (1:20; 17251-1-AP) and type XI collagen from Santa Cruz Biotechnology (1:20; sc-74372) were detected using donkey anti-mouse IgG conjugated to Alexa Fluor-647 (1:500), donkey anti-rabbit IgG conjugated to Alexa Fluor-594 (1:500) and donkey anti-goat IgG conjugated to Alexa Fluor-488 (1:500) from Invitrogen, respectively. Co-immunolabellings by antibodies against type I collagen from Abnova (1:20; H00001277-M01), type VIII collagen from Santa Cruz Biotechnology (1:20; sc-99356) and ACTA2 from Abcam (1:100; ab5694) were detected using donkey anti-mouse IgG conjugated to Alexa Fluor-647 (1:500), donkey anti-goat IgG conjugated to Alexa Fluor-488 (1:500) and donkey anti-rabbit IgG conjugated to Alexa Fluor-594 (1:500), respectively. Co-immunolabellings by antibodies against type XI collagen from Santa Cruz Biotechnology (1:20; Sc-74372) and F4/80 from Abcam (1:100; ab6640) were detected using donkey anti-goat IgG conjugated to Alexa Fluor-488 (1:500) and goat anti-rat IgG conjugated to Alexa Fluor-594 (1:500), respectively. Nuclei were visualized by mounting the cells in DAPI-containing Vectashield mounting medium. Multi-labelled cells were visualized using the Leica TCS SP8 STED 3X confocal microscope.

### Statistical analysis

Data are expressed as mean±standard deviation (s.d.). For pairwise comparison of fold mRNA expression between untreated and TGFβ2-treated cells for each collagen gene, the significance of differences between the two conditions was determined using the two-tailed Student's *t*-test. For comparison of fold mRNA expression in operated tissues between different time points or of densitometric analyses where fold protein expression between unoperated and operated tissues or between untreated and TGFβ2-treated cells were compared, the significance of differences between the time points or conditions across the three collagen proteins, was determined using one-way ANOVA with Bonferroni post-hoc adjustment using SPSS statistics. *P*<0.05 was deemed to be significant.
